# Prevalence of overweight and obesity and some associated factors among adult residents of northeast China: a cross-sectional study

**DOI:** 10.1136/bmjopen-2015-010828

**Published:** 2016-05-05

**Authors:** Rui Wang, Peng Zhang, Chunshi Gao, Zhijun Li, Xin Lv, Yuanyuan Song, Yaqin Yu, Bo Li

**Affiliations:** Department of Epidemiology and Biostatistics, Jilin University School of Public Health, Changchun, Jilin, China

**Keywords:** overweight, obesity, influencing factors, northeast China

## Abstract

**Objectives:**

This study aims to estimate the prevalence of overweight and obesity and determine potential influencing factors among adults in northeast China.

**Methods:**

A cross-sectional survey was conducted in Jilin Province, northeast China, in 2012. A total of 9873 men and 10 966 women aged 18–79 years from the general population were included using a multistage stratified random cluster sampling design. Data were obtained from face-to-face interview and physical examination. After being weighted according to a complex sampling scheme, the sample was used to estimate the prevalence of overweight (body mass index (BMI) 24–27.9 kg/m^2^) and obesity (BMI >28 kg/m^2^) in Jilin Province, and analyse influencing factors through corresponding statistical methods based on complex sampling design behaviours.

**Results:**

The overall prevalence of overweight was 32.3% (male 34.3%; female 30.2%), and the prevalence of obesity was 14.6% (male 16.3%; female 12.8%) in Jilin Province. The prevalence of both overweight and obesity were higher in men than women (p<0.001). Influencing factors included sex, age, marriage status, occupation, smoking, drinking, diet and hours of sleep (p<0.05).

**Conclusions:**

This study estimated that the prevalence of overweight and obesity among adult residents of Jilin Province, northeast China, were high. The results of this study will be submitted to the Health Department of Jilin Province and other relevant departments as a reference, which should inform policy makers in developing education and publicity to prevent and control the occurrence of overweight and obesity.

**Strengths and limitations of this study**

- The main strengths of this study are its large sample size and precise physical measurement, which improved the validity of the results.
- Some potential limitations existed in this cross-sectional study. Self-reported data and the nature of cross-sectional data may lead to recall and reporting biases, which may have affected the accuracy of the results.
- The results were from Jilin Province only and therefore cannot be generalised to the whole northeast of China.

## Introduction

Overweight and obesity have been shown to be related to multiple chronic conditions, and lead to a heavy economic burden on families and increasing costs to society throughout the world.^1–3^ According to a WHO report,^4^ obesity is defined as a body mass index (BMI) ≥30 kg/m^2^, and overweight as a BMI of 25–29.9 kg/m^2^. For Chinese people, obesity suggests a BMI ≥28 kg/m^2^ and overweight indicates a BMI of 24–27.9 kg/m^2^.^5^ In 2013, in order to make physicians pay more attention to the condition, the American Medical Association classified obesity as a disease.^6^

China is the largest developing country and has the largest population in the world. Its rapid economic growth and enviable developmental performance have contributed to changes in lifestyle such as dietary habits and physical activity.^7^ Over the past few decades, China has witnessed an obesity epidemic which has led to various diseases, especially chronic ones. Overweight and obesity have become a major public health problem in China, although the prevalence of obesity is lower than in developed countries.^4^ ^8^ ^9^ Previous research suggests that there has been a significant increase in the prevalence of obesity in the USA and Canada over the past few years,^10^ and the same scenario as has played out in China recently, and there is no doubt that the rapidly increasing occurrence of overweight and obesity in China will continue to increase the prevalence of chronic diseases.^11–13^ Therefore, understanding influencing factors associated with overweight and obesity will be useful for policy makers in formulating policies to diminish the rate and to control related comorbid conditions.

Jilin Province is located in northeast China, and has a large population of about 27 million.^14^ In 2012, Jilin Department of Health and Jilin University jointly conducted the Jilin Provincial Chronic Disease Survey, which is the first large representative population-based survey of chronic disease in this area. Data used in our study were obtained from this survey. In this article, we estimate the prevalence of overweight/obesity and explore potential influencing factors in adult residents of northeast China. The result of this study will be considered as a reference for policy makers in making informed decisions.

## Methods

### Study design and population

This population-based cross-sectional survey is part of the Project on Present Situation and Change Forecast of Disease Spectrum in Jilin Province of China in 2012. Face-to-face interviews on health and physical examinations were performed for the Jilin Provincial Chronic Disease Survey in residents aged 18–79 years; all the subjects included had lived in Jilin Province for more than 6 months. The sample size calculation of this survey was used to estimate the prevalence of overweight and obesity in Jilin Province. The ultimate target sample size was established to be 25 240, accounting for about 1‰ of the total adult population of Jilin Province.

We used the multistage stratified cluster sampling method to select the study sample. First, we stratified Jilin Province into nine regions (Changchun, Jilin (city), Siping, Liaoyuan, Tonghua, Baishan, Songyuan, Baicheng and Yanbian) covering the whole of Jilin. These regions are all primarily responsible for the administration of healthcare services. Then, from each of the nine regions, we randomly selected clusters of four districts or counties. Thirty-two districts or counties, 95 towns or communities, and 45 units were selected. Finally, each participant was selected randomly from each household in the sites mentioned above.^14^ ^15^

### Data collection

The formal survey was launched on 5 July 2012 and lasted 34 days. It was made up of two parts: face-to-face interview and physical examination. Before the formal survey, we conducted a pre-survey to explore the feasibility of the questionnaire. After the pre-interview, 116 uniformly trained investigators conducted the face-to-face interviews and physical examinations at local health centres or community clinics. All the participants' identities were confirmed by the investigators. During the investigation, each completed questionnaire was examined by two investigators to ensure validity and consistency. After the fieldwork, data were manipulated by parallel double entry, and we also performed three verifications to check for incomplete and inconsistent responses.

The questionnaire provided demographics, lifestyle habits and other related information on health. Height and weight were determined using a standardised protocol with the subjects in light indoor clothing without shoes. Height was measured to the nearest 0.1 cm, and weight to the nearest 0.1 kg.

### Definition of variables

According to the National Bureau of Statistics of China, each selected district or county in this survey was divided into urban and rural areas.^16^ The grading standards for a Chinese adult have been mentioned above: BMI=weight/height^2^ (kg/m^2^); 24≤BMI<28 is overweight; BMI≥28 indicates obesity; we used the criterion BMI≥24 as the assessment standard of excessive weight. Education was classified into four levels: primary school and below (including those who had never attended school and those with elementary schooling only); junior school; high school (including those with 3 years of secondary vocational schooling); undergraduate and above levels of education. Manual labour included farmers, service workers and production workers. White collar occupations included office and other technical employment. Other occupations included student, unemployed, full-time housewife and retiree.^14^ We defined a smoker as a person who had smoked at least one cigarette a day over the past 30 days. A drinker was defined as a person who had consumed more than one alcoholic drink a week, including any form of alcohol. Participants who were classified as ‘eating more meat’ were defined as persons who had eaten more animal-based foods than vegetables during meals in the past 30 days. A ‘balanced diet’ refers to eating animal-based foods and vegetables in equal measure during meals in the past 30 days. Participants who were classified as ‘sleep <7 h’ were defined as persons who slept less than 7 h over 3 days a week, and those who slept more than 9 h over 3 days a week were defined as ‘sleep >9 h’.

### Statistical analysis

We used post-stratification adjustment according to the distribution of regional, urban/rural, age and sex groups in the 2010 census of the adult population of Jilin Province to make the sample estimate of the population of the whole province. Frequency distribution was used to present characteristics of the subjects, and data presented as percentages were used to report the prevalence ratio. Rao–Scott χ^2^ tests were used to compare the prevalence of overweight and obesity in different groups. To adjust for potential confounding effects, multiple regression analyses were carried out to explore independent factors associated with overweight and obesity. OR with 95% CI was used for the risk analysis. All statistical analyses were conducted using the complex sampling function of SPSS V.20.0, and a p≤0.05 level of significance was selected. The map was drawn by MapInfo Professional V.7.0 software.

### Ethics approval

This study was approved by the Ethics Committee of Jilin University School of Public Health (reference number 2012-R-011), and written informed consent was obtained from all subjects in the survey.

## Results

**Figure 1:**
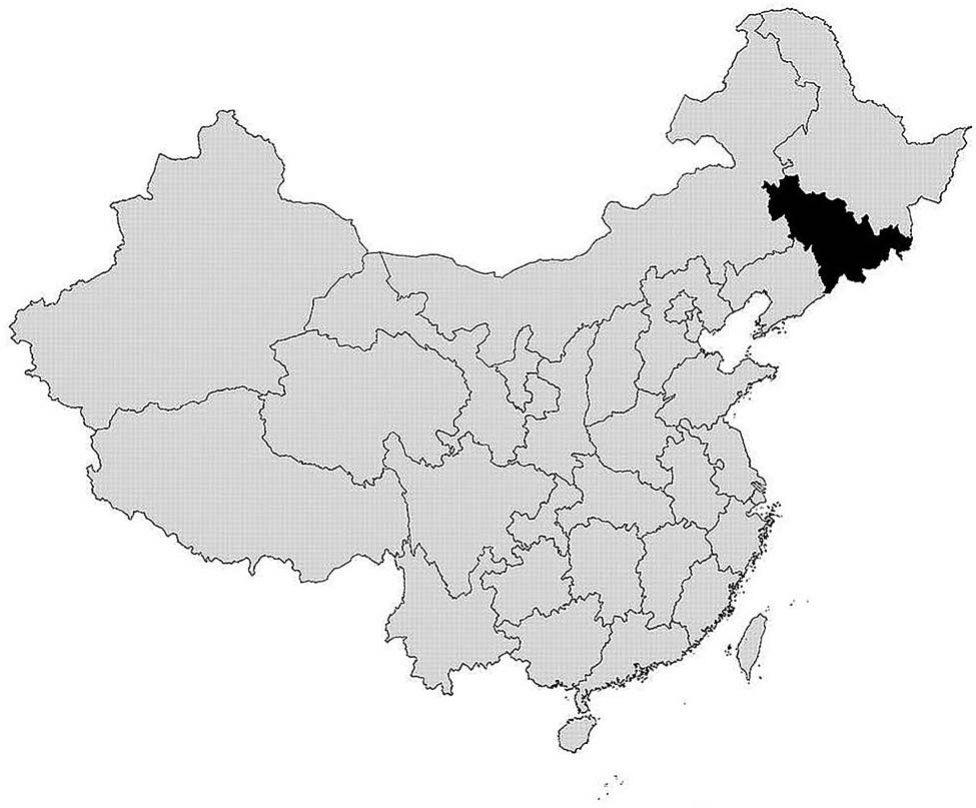
Geographical position of Jilin Province in northeast China.

In this survey, we interviewed 23 050 residents aged over 18 years; 2211 were excluded because of lack of information and other potential bias, leaving 20 839 in the final analysis, a response rate of 82.6% (replacement rate 9.5%). Of the 20 839 subjects, 9873 (47.4%) were men and 10 966 (52.6%) were women; ages ranged from 18 to 79 years (mean±SD 47.27±13.34) (table 1). According to the BMI classification for Chinese people, the overall prevalence of overweight was 32.3% (male 34.3%; female; 30.2%), and the prevalence of obesity was 14.6% (male 16.3%; female 12.8%) in Jilin Province. Figure 1 shows the geographical position of Jilin Province in northeast China.

**Table 1:** Prevalence of overweight and obesity according to demographic characteristics

		Overweight			Obesity		
Characteristic	n	PR (%)	χ^2^	p Value	PR (%)	χ^2^	p Value
Area							
Urban	10 733	32.9	4.25	0.088	14.6	<0.001	0.996
Rural	10 106	31.6			14.6		
Sex							
Male	9873	34.3	40.58	<0.001	16.3	50.82	<0.001
Female	10 966	30.2			12.8		
Age							
18–24	1128	14.5	694.27	<0.001	7.8	130.16	<0.001
25–34	2787	27.1			15.8		
35–44	4749	34.3			15.5		
45–54	5627	39.8			16.5		
55–64	4483	40.5			15.7		
65–79	2065	37.0			13.9		
Education							
Primary school and below	6143	34.5	27.81	<0.001	15.3	7.79	0.171
Junior middle school	5977	31.1			15.1		
Senior middle school	5349	33.5			14.1		
Undergraduate and above	3370	30.1			13.6		
Marriage							
Unmarried	1569	16.7	393.12	<0.001	10.2	56.95	<0.001
Married	17 861	34.8			15.4		
Separated/divorced/widowed	1409	37.4			14.1		
Occupation							
Manual	11 689	32.0	3.09	0.364	14.2	33.61	<0.001
White collar	4078	32.1			12.9		
Other	5072	33.4			17.0		

Complex weighted computation was used in the statistical analysis.

PR, prevalence rate.

From the age-stratified results in table 2, for men, the prevalence of overweight increased with age, peaking at 45–54, while no such trend was seen in the prevalence of obesity. For women, the prevalence of both overweight and obesity increased with age, peaking at 55–64 and 65–79, respectively. Moreover, the prevalence of overweight was higher in men than women in the age groups 18–54, and the prevalence of obesity was higher in men than women in the age groups 18–44.

**Table 2:** Prevalence of overweight and obesity in men and women according to age group

	Overweight			Obesity		
Age	Male (%)	p Value	Female (%)	Male (%)	Female (%)	p Value
18–24	18.0	11.1	<0.001	11.4	4.2	<0.001
25–34	31.6	21.3		21.1	8.9	
35–44	37.9	30.6		18.5	12.3	
45–54	41.0	38.5		15.9	17.2	
55–64	39.5	41.6		13.8	17.6	
65–79	33.9	40.0		9.8	17.7	
Total	34.3	30.2		16.3	12.8	

We divided the participants into two groups: normal; overweight and obese. Table 3 shows that the following factors all had a significant effect: sex, age, education, marriage status, occupation, smoking, drinking, diet and sleep quality (p<0.001). We therefore added all these significant factors to a multivariate logistic regression model.

**Table 3:** Univariate analysis of correlates of overweight and obesity in residents of Jilin Province

					95% CI for OR	
Characteristic	PR (%)	Wald χ^2^	p Value	OR	Lower	Upper
Area		2.39	0.122			
Urban	47.5			1.00	–	–
Rural	46.2			0.95	0.89	1.02
Sex		78.15	<0.001			
Female	43.0			1.00	–	–
Male	50.6			1.36	1.27	1.45
Age		77.41	<0.001			
18–24	22.4			1.00	–	–
25–34	42.9			2.60	2.16	3.14
35–44	49.8			3.44	2.89	4.10
45–54	56.3			4.47	3.76	5.31
55–64	56.2			4.46	3.74	5.32
65–79	50.9			3.59	2.95	4.38
Education		8.25	<0.001			
Primary school and below	49.8			1.00	–	–
Junior middle school	46.2			0.86	0.79	0.94
Senior middle school	47.6			0.92	0.84	1.00
Undergraduate and above	43.7			0.78	0.70	0.87
Marriage		101.71	<0.001			
Unmarried	26.9			1.00	–	–
Married	50.2			2.74	2.39	3.15
Separated/divorced/widowed	51.2			2.85	2.38	3.42
Occupation		9.23	<0.001			
Manual	46.2			1.00	–	–
White collar	45.0			0.95	0.87	1.04
Other	50.4			1.18	1.08	1.29
Smoking		53.37	<0.001			
No	46.0			1.00	–	–
Yes	45.2			0.97	0.90	1.04
Ever	61.4			1.86	1.65	2.11
Drinking		32.99	<0.001			
Yes	50.4			1.00	–	–
No	45.1			0.81	0.75	0.87
Diet		24.06	<0.001			
Balanced	46.7			1.00	–	–
More meat	56.0			1.45	1.28	1.65
More vegetables	44.4			0.91	0.85	0.98
Sleep (hours/night)		33.51	<0.001			
<7	51.6			1.00	–	–
>7	45.2			0.77	0.72	0.83
>9	41.8			0.68	0.60	0.76

PR, prevalence ratio.

Table 4 shows the results of logistic regression models comparing the prevalence of the potential risk factors: sex, age, level of education, marriage status, occupation, smoking, drinking, diet and hours of sleep. The multivariate logistic regression results reveal that male adults are more likely to become overweight and obese than female adults (OR 1.50, 95% CI 1.37 to 1.65). We categorised age into six groups, which clearly showed that increasing age is a risk factor for overweight/obesity. Participants who have married (OR 1.44, 95% CI 1.19 to 1.74) or are separated/divorced/widowed (OR 1.44, 95% CI 1.14 to 1.82) are more likely to be overweight/obese than those who have never married. In addition, cigarette smokers (OR 0.70, 95% CI 0.64 to 0.77) are less likely to become overweight and obese than non-smokers and ever-smokers (OR 1.23, 95% CI 1.07 to 1.40). Participants who drink are more likely to become overweight or obese than those who never or rarely drink (OR 1.11, 95% CI 1.02 to 1.21). Compared with people on a balanced diet, those who eat more meat (OR 1.47, 95% CI 1.29 to 1.68) are more likely to put on weight than those who eat more vegetables (OR 0.83, 95% CI 0.77 to 0.89). Overweight and obesity are more common among those who sleep <7 h compared with those who sleep >7 h (OR 0.89, 95% CI 0.82 to 0.96) and >9 h (OR 0.83, 95% CI 0.74 to 0.94).

**Table 4:** Multivariate regression analysis of correlates of overweight and obesity in residents of Jilin Province

						95% CI for OR	
Characteristic	Wald χ^2^	p Value	β	SE	OR	Lower	Upper
Sex							
Female	–	–	–	–	1.00	–	–
Male	75.04	<0.001	0.41	0.05	1.50	1.37	1.65
Age							
18–24	–	–	–	–	1.00	–	–
25–34	47.71	<0.001	0.79	0.11	2.20	1.76	2.75
35–44	79.05	<0.001	1.05	0.12	2.86	2.27	3.61
45–54	118.0	<0.001	1.30	0.12	3.65	2.89	4.61
55–64	101.00	<0.001	1.23	0.12	3.44	2.70	4.37
65–79	49.60	<0.001	0.95	0.14	2.60	1.99	3.38
Education							
Primary school and below	–	–	–	–	1.00	–	–
Junior middle school	2.76	0.097	−0.08	0.05	0.92	0.84	1.02
Senior middle school	1.04	0.308	−0.05	0.05	0.95	0.86	1.05
Undergraduate and above	0.01	0.921	0.01	0.07	1.01	0.88	1.15
Marriage							
Unmarried	–	–	–	–	1.00	–	–
Married	13.60	<0.001	0.36	0.10	1.44	1.19	1.74
Separated/divorced/widowed	9.50	0.002	0.37	0.12	1.44	1.14	1.82
Occupation							
Manual	–	–	–	–	1.00	–	–
White collar	0.59	0.443	−0.04	0.06	0.96	0.86	1.07
Other	26.16	<0.001	0.25	0.05	1.28	1.17	1.41
Smoking							
No	–	–	–	–	1.00	–	–
Yes	56.49	<0.001	−0.35	0.05	0.70	0.64	0.77
Ever	8.96	0.003	0.21	0.07	1.23	1.07	1.40
Drinking	5.33	0.021	0.10	0.05	1.11	1.02	1.21
Diet							
Balanced	–	–	–	–	1.00	–	–
More meat	33.30	<0.001	0.39	0.07	1.47	1.29	1.68
More vegetables	23.53	<0.001	−0.19	0.04	0.83	0.77	0.89
Sleep (hours/night)							
<7	–	–	–	–	1.00	–	–
>7	9.67	0.002	−0.12	0.04	0.89	0.82	0.96
>9	8.64	0.003	−0.18	0.06	0.83	0.74	0.94
Constant	83.78	<0.001	4.96	0.46	–	–	–

## Discussion

As far as we know, this is the first large population-based face-to-face survey to investigate the prevalence of overweight and obesity in Jilin Province, northeast China. We found that the prevalence of both overweight and obesity was high. From data from the China Chronic Disease Survey conducted by the Chinese Center for Disease Control and Prevention, the prevalence of overweight among Chinese adults (age 18–64) for 2004, 2007 and 2010 was 23.8%, 26.6% and 30.6%, respectively (male 23.0%, 27.4% and 32.1%; female 24.7%, 25.7% and 29.1%), and the prevalence of obesity was 7.2%, 7.7% 12.1%, respectively (male 6.3%, 6.7% and 12.5%; female 8.1%, 8.7% and 11.1%).^17^ Our cross-sectional study indicates that the prevalence of overweight and obesity among adults in northeast China was 32.3% (male 34.3%; female 30.2%) and 14.6% (male 16.3%; female 12.8%), respectively, in 2012, which is relatively low compared with developed countries.^1^ ^18^ However, with the rapid economic development of recent years, dietary habits in China have gradually changed from oriental to Western.^19^ ^20^ Compared with the prevalence of overweight and obesity at national level, this study shows a higher prevalence in both sexes. This implies that overweight and obesity are more common in northeast China than in other areas, although effective actions might have been taken to control the upward trend.^21–23^

Multivariate logistic regression analysis suggested that several factors are associated with the prevalence of overweight and obesity. The prevalence of overweight is higher in men than women from the age of 18 to 54, and there is a higher prevalence of obesity from the age of 18 to 44, which is in line with previous studies that reported sex difference associated with BMI.^24^ For women, the prevalence of both overweight and obesity increased with age, peaking at 55–64 years. A possible explanation is changes in hormone levels with age.^25^ ^26^

One review from the USA revealed that different marital transitions are associated with changes in body weight for both sexes: transition into marriage appears to be associated with weight gain, whereas transition out of marriage is associated with weight loss.^27^ The present study found the slightly different result that, in adults in northeast China, both transition into marriage and out of marriage are risk factors for becoming overweight and obese. This may be due to racial differences, but the exact mechanism is not clear; further research is needed to illustrate this apparent association with marriage status.

Besides the demographic characteristics mentioned above, behaviours and lifestyle are also found to be associated with body weight. In this study, we found a lower prevalence of overweight and obesity in smokers than non-smokers, which is consistent with studies conducted in India, Switzerland and Tianjin, China.^28–30^ As many studies have reported previously, tobacco use is associated with various chronic diseases and body disorders, which may affect the digestive and absorptive function of the alimentary system.^31^ ^32^ Furthermore, some research has also suggested that smoking increases energy expenditure and might suppress appetite.^33^ In addition, it has been suggested that some young people use smoking as a method of weight control; they smoke for the purpose of losing weight.^34^ ^35^ As the use of tobacco does more harm than good, we really do not recommend this way of losing weight.

To the best of our knowledge, diet is one of the most important determinants of body weight. We have observed that drinking and eating more meat are risk factors for being overweight and obese, which is consistent with previous studies.^30^ Animal-based foods contain more fat than other foods, and regular consumption of high-fat foods will lead to weight gain. Affected by the traditional culture of drinking alcohol, especially in northeast China, it is generally believed that, the more you drink, the more emotion you invest in your friends and relatives. Wine is not drunk when people get together.

Recent studies have shown that poor sleep quality significantly affects BMI.^36^ ^37^ The results of the present study support this conclusion. A plausible explanation is that sleep disturbances rather than sleep duration may contribute to overweight and obesity, but further studies of the biological mechanisms are needed.

Education level, occupation and area of residence had no significant effect on body weight. Because we used a cross-sectional design in this study, our ability to determine causal inference is limited, and therefore we are unable to explain why these factors are associated or not with overweight and obesity. Further research is needed to better understand why these associations occur, why the observed disparities exist, and how to reverse these trends.

The results of this study form a significant part of the Disease Spectrum in Jilin Province of China, which will be submitted to the Health Department of Jilin Province and other relevant departments as a reference. These departments should heed the high prevalence of overweight and obesity in Jilin Province and provide effective guidelines to help to reverse the trend.

The main strengths of our study are its large sample size and precise physical measurements, which increase the validity of our results. However, some potential limitations exist in this cross-sectional study. Self-reported data and the nature of cross-sectional data may lead to recall and reporting biases, which may have affected the accuracy of the results. The results of this study were from Jilin Province only, and cannot be generalised to the whole of northeast China. In addition, we excluded the item ‘exercise’, which has always been considered an important factor in overweight and obesity. During the process of data collection, we found ‘exercise’ was related to the item ‘occupation’, which may have a large impact on the results. More than 30% of the participants were manual workers, and they claimed that the heavy manual labour they performed left them with no energy to take extra exercise, which resulted in most of the answers to the item about ‘exercise’ being ‘rarely or never’. However, their actual energy consumption should be high. The same was true for full-time housewives because of the housework they performed, especially in rural areas.

## Conclusions

To sum up, this study suggests that the prevalence of overweight and obesity among adult residents of Jilin Province, northeast China, is high. The main influencing factors for overweight and obesity are sex, age, marriage status, occupation, smoking, drinking, dietary habits and sleep quality.

The results of this study will be submitted to the Health Department of Jilin Province and other relevant departments as a reference, which should inform policy makers in developing education and publicity to prevent and control the occurrence of overweight and obesity.
